# Nasal and Pharyngeal Manifestation of Syphilis—Result and Treatment

**Published:** 1891-01

**Authors:** J. G. Carpenter

**Affiliations:** Stanford, Ky.


### NASAL AND PHARYNGEAL MANIFESTATION OF SYPHILIS.—RESULT AND TREATMENT.*

BY J. G. CARPENTER, M. D., STANFORD, KY.

Primary syphilis, including the period of inoculation, development of the chancre and infection of the adjacent glands, is found infrequently in the nasal and pharyngeal chambers, though more frequently in the mouth and upon the lips, consequently the secondary form of syphilis and its sequelæ concerns the rhinologist more than any other variety.

What was once considered tertiary and quarternary syphilis by former syphilographers is to-day, in presence of scientific light, called sequelæ of syphilis.

All modern aggressive specialists in venereal diseases admit there is a contagium of syphilis, and that it is a self-limited disease and primarily infects the system through the lymphatic

*Read before the American Rhinological Association, Louisville, Ky., October 6th, 1890.

channels and does not contaminate the blood until the chyle is emptied from the thoracic duct into the left subclavian vein, when poisoning of the blood and general infection of the system take place. Dr. Ephraim Cutter, of New York City, claims to have discovered and isolated the contagium of syphilis; viz., the "crypta syphilitica." Other scientists claim otherwise, and the question is yet "*sub judice*," unless Dr. Cutter's views are corroborated. In Gaillard's medical journal, 1884, there is this statement made by Dr. Cutter. There are some physicians who are familiar with the morphology of the blood of syphilitics. By microscopically inspecting the blood of a syphilitics, they make out the diagnosis of the disease at once with a certitude unusual in human affairs. Besides this, they tell when the patient is really cured by noting the disappearance from the blood of the peculiarities of syphilitic blood and claim the "*syphilitic*" can transmit syphilis so long as the *crypta syphilitica* are found in the blood. It is claimed for the latter that algoid forms of vegetable life exist; the white corpuscles are enlarged and contain copper-colored spores and filaments, with obtusely rounded ends; these spores and filaments are found in the primary sores (chancres), in the pus and secretions of secondary lesions. The presence of syphilis is signalized by local cell proliferation, causing salient engorgement and induration at the point of infection; then the adjacent glands, finally every recognized lesion of syphilis. The juices or secretions of any of these lesions will produce syphilis in a healthy person. Otis states these secretions contain, microscopically, cell material germinal matter; so far, it is impossible to distinguish the diseased cells from the healthy. It is only by its abnormal amount and peculiar aggregation, together with its infectial property, that it can be differentiated from the most healthy germinal matter. The initial lesions of syphilis, microscopically, show the indurations are made up of cell accumulations, which involve the walls of the blood vessels, even obliterating them, and indicates it is not an inflammatory process, but a local cell proliferation. In the induration of syphilis there is a dry anemic tissue, resistant connective tissue fibers, considerably thickened walls of the vessels, which make it difficult for the serum to escape, and also diminishes the calibre of the vessels, and this explains why the syphilitic induration breaks down into a molecular mass, and why resorption takes place so slowly ; the cell accumulations which constitute this induration have been found to occur in the lymphatic vessels in communication with it and recognized like knotty cords under the integument, running to the lymphatic glands, into which they empty and in time become depots for the proliferative process, and are proven to be characteristic of the presence and advance of syphilitic disease. That only during the active stages of syphilis, the primary and secondary periods, the contagious element of syphilis is present ; therefore, hereditary transmission of the disease is only possible during this time, usually one to three years.

The sequelæ of syphilis (the tertiary and quarternary periods) contain no discernible element of contagion ; microscopic examination of the various forms of germata, including the eruptions, have failed to demonstrate anything besides the fibers of normal germinal elements. Repeated inoculations of these products have failed to disclose any contagious principle ; without contagion there is no syphilis. The physiological secretions, milk, saliva, urine, perspiration, tears and spermatic fluid have not been proven to be agents of syphilitic infection.

The presence of the initial lesions of syphilis in the nasal or pharyngeal chambers causes swelling of the mucous lining, pain, fever, infection of adjacent glands and in the nasal chambers difficult nasal respiration.

Syphilitic rhinitis may occur as the result of heredity; the local lesions of secondary syphilis found in the upper air passages may begin as catarrhal inflammation (rhinitis or pharyngitis), a local circumscribed erythema, a papular, pustular or tubercular deposit; the epithelium of the mucous membrane becomes rapidly softened, disintegrated and abraded, leaving one or several erosions, becoming quite painful, sensitive and at first bathed in a mucous secretion, which soon becomes muco-purulenti, or purulent as the ulcers enlarge and disintegrate the secretion, and pain increases and the erythematous area becomes more extensive, is puffy and more livid than normal; the surface of the ulcers presents a dirty ashy gray aspect and daily gets more excavated and is circular, oblong or irregular in shape. If the ulcerative process is not checked by appropriate treatment, the submucous tissue and even muscular tissue get involved; the destructive process may reach the periosteum and cartilage. The secretion is now a yellow or yellowish green, the aqueous element evaporates, inspissation and desiccation of the secretion result, with the formation of scabs or brown crusts, streaked with blood and mixed with disintegrated tissue. The secretions and crusts hang tenaciously and give a very offensive odor, and act as a constant source of irritation to air passages ; necrosis of the septal and laryngeal cartilages, and adjacent bony walls, which make up the frame or skeleton of the upper air passages, occurs as the sequelæ of secondary syphilis, and occurs five to thirty years after the initial lesion has been received, and to the writer it seems in those who have been improperly treated or neglected.

Those syphilitics who have been treated one year and a-half to four years by an expert have remained free and proof against tertiary manifestations. This hasty proliferation of syphilis, like normal cell material in the excess of the necessities of growth and repair, undergoes fatty metamorphosis, and is eliminated from the effete organism. Braumler states this takes place in the majority of cases, and at the expiration of eighteen months or two years the infection is entirely exhausted. In treating syphilis of the nose and throat, it is very important to arrest and cure the disease in the shortest period possible to prevent destruction of tissue and deformity.

Following syphilitic ulceration of the soft tissue of the nose palate, pharynx and larynx, are deformity, dysphagia, and faulty voice ; the anterior or posterior nares or Eustachian orifices may be occluded by adhesion, so as to interfere with their function ; the oro-nasal aperture may become obstructed by partial or complete adhesions of the uvula or palate to the posterior wall of the pharynx; the anterior and posterior palatine walls on one or both sides may be destroyed, also the palate in part or totally ; the destructive lesions may extend by continuity of tissue from lower wall of pharynx, destroying the epiglottis, the vocal cords and mucous membrane of larynx. The nose and its bones may be destroyed. It is in the tertiary lesions or sequelæ of syphilis that such destructive lesions of the soft parts with caries and necrosis of bones and cartilage result. Stenosis from cicatricial deformity may result in ulceration of larynx and necessitate tracheotomy.

What is often differentiated as gummata or syphilomata of the bones of the nasal and pharyngeal chambers is disintegrated soft tissue leading to dead bone.

Goodwillie states necrosis of the bones of the internal nose and the maxillæ occur in the following order; viz., the vomer, vault of hard palate, lower portion of the ethmoid, inferior turbinated, the inter-maxillary, the turbinated, the antrum wall, the maxillæ and the superior turbinated. The best remedy for the cure and treatment of syphilis is mercury ; it produces fatty metamorphosis of living tissue. Healthy persons quickly emaciate, and all kinds of tissue break down under its continuous excessive use. All remedies, which have aided in the cure of syphilis, have caused fatty metamorphosis of tissue.

The grand object to be accomplished in the treatment of syphilis is to arrest and eliminate superfluous cell proliferations and thereby eliminate the contagium of syphilis. The continuous use of mercury in small tonic doses from one to three years, short of producing tenderness of the gums and slight ptyalism or relaxation of the bowels, is the most appropriate treatment for the permanent cure and eradication of the disease from the system.

The proto-iodide, the biniodide and the bichloride of mercury stand at the head of mercurial preparations. External applications to the groins, arm-pits, inside of thighs, two or three times daily, are adjuvants of the greatest importance in the treatment of syphilis.

Whatever constitiutional vices or functional derangements exist must be corrected. The old maxim, "that we treat the patient and not the disease," must not be forgotten in the treatment of syphilis. No two obstacles stand more in the way of a successful issue in treatment than the use of rum and tobacco. The use of them must be restrained, as otherwise a tedious convalescence can only be anticipated.

Otis states a judicious mercurial treatment will hasten the cure of the acute lesions of syphilis; but it is not on this account it is so essential, but the fact that it prevents the sequelæ, which results in destruction of important tissues and organs, vessels and bones. In some cases the mixed treatment is preferred, from the first; in the last half of the treatment it is doubtless best to use it. After a long mercurial course it is judicious to give the iodide of potash, pushed to the point of physiological toleration, each patient being a law unto himself as to size and frequency of dosage; the iodide of potash stands next to mercury in causing fatty metamorphosis of tissue, and is highly essential in the secondary lesions, as well as in the *sequelæ*, and should be given in the largest dose, short of iodism. (Pushed too far it impoverishes the blood, diminishes the quantity of fibrin, increasing waste and consequent tissue metamorphosis, interfering with the function of nutrition and producing a condition of cachexia, with catarrhal secretions.—GOODWILLIE.)

LOCAL TREATMENT.

The local treatment of syphilitic lesions of the nose, pharynx and larynx consists in mild antiseptic and aseptic soothing, cleansing application to disinfect, soften, detach and wash away the morbid secretion and crusts which vitiate the respired air and act as foreign material, causing constant irritation, blowing and hawking, and to use such other medicaments as will arrest and restore the affected parts to a normal state in the shortest period, in conjunction with constitutional measures.

Listerine, ʒi–iii; glycerine, ℥ii; salt, grs. v–x. Mix. Listerine, ʒii; aqua, ℥x; sodii bicarbonate, sodii biborate, ää grs. v. Mix. Or, salt, ʒi; aqua, ℥xvi; snuffled through the nose, with the head in three positions, and gargled in the throat, or sprayed with a DeVrebis spray tube, will usually cleanse the parts thoroughly, though several sittings have to be made in some cases before thorough cleansing is accomplished.

If mucous patches or superficial ulcers exist, mild caustic applications must be made, as the case demands ; if ulcers are deep-seated, carbolic or nitric acid, the acid nitrate of mercury, or solid stick of silver must be applied, or the cautery, due care being taken to protect sound tissue. After thorough cleansing and cauterizing, it will be advisable to dust the sores with iodoform, bismuth or tannin, or apply campho-phenique. Should a chancre be present, it requires the same treatment and attention as in other regions. For the syphilitic rhinitis, pharyngitis or laryngitis, after cleansing is accomplished, if the inflammation and pain is very intense, muriate of cocaine, 2% or 4% salt, should be sprayed, followed by vaseline, ℥i ; oil of eucalyptus, gtts. v ; menthol, grs. iii. Mix. Or, menthol and vaseline in the same proportion ; or, iodol, grs. x; vaseline, ℥i; sprayed upon the affected parts. Acidi carbol., grs. v; Kennedy's pinus canadensis ʒii–iv; aqua, ℥vi; glycerine, ℥iss, is a most excellent tonic and astringent application, and will, in many cases, be more essential than other measures.

In the removal of the tenacious secretions, the probe and forceps may be in demand.

For the treatment of the sequelæ of syphilis, caries and necrosis of bone and cartilage of the contiguous bones of the nasopharynx and larynx, the same surgical rules hold good as in other localities, viz., to remove the dead bone, prevent further disintegration and render the wound aseptic. Dr. Goodwillie conferred a blessing upon rhinologists when he invented the circular revolving single and multiple knives and saws and drills for removing dead bone from the walls of the upper air passages. Attached to the dental engine, they remove the sequestra very quickly. While local measures are essential, constitutional treatment is the one thing needful in removing the dyscrasia.

